# The effect of water temperature on orthostatic tolerance: a randomised crossover trial

**DOI:** 10.1007/s10286-022-00860-7

**Published:** 2022-04-23

**Authors:** Iain T. Parsons, Brooke C. D. Hockin, Omnia M. Taha, Natalie D. Heeney, Erin L. Williams, Vera-Ellen M. Lucci, Rebekah H. Y. Lee, Michael J. Stacey, Nick Gall, Phil Chowienczyk, David R. Woods, Victoria E. Claydon

**Affiliations:** 1 Research and Clinical Innovation, Royal Centre for Defence Medicine, Birmingham, UK; 2 School of Cardiovascular Medicine and Sciences, King’s College London, London, UK; 3 Department of Biomedical Physiology and Kinesiology, Simon Fraser University, Burnaby, BC Canada; 4 Department of Surgery and Cancer, Imperial College London, London, UK; 5 King’s College Hospital, London, UK; 6 Carnegie School of Sport, Leeds Beckett University, Leeds, UK

**Keywords:** Syncope, Orthostatic tolerance, Bolus water drinking, Cardiovascular control

## Abstract

**Purpose:**

Bolus water drinking, at room temperature, has been shown to improve orthostatic tolerance (OT), probably via sympathetic activation; however, it is not clear whether the temperature of the water bolus modifies the effect on OT or the cardiovascular responses to orthostatic stress. The aim of this study was to assess whether differing water temperature of the water bolus would alter time to presyncope and/or cardiovascular parameters during incremental orthostatic stress.

**Methods:**

Fourteen participants underwent three head-up tilt (HUT) tests with graded lower body negative pressure (LBNP) continued until presyncope. Fifteen minutes prior to each HUT, participants drank a 500 mL bolus of water which was randomised, in single-blind crossover fashion, to either room temperature water (20 °C) (ROOM), ice-cold water (0–3 °C) (COLD) or warm water (45 °C) (WARM). Cardiovascular parameters were monitored continuously.

**Results:**

There was no significant difference in OT in the COLD (33 ± 3 min; *p* = 0.3321) and WARM (32 ± 3 min; *p* = 0.6764) conditions in comparison to the ROOM condition (31 ± 3 min). During the HUT tests, heart rate and cardiac output were significantly reduced (*p* < 0.0073), with significantly increased systolic blood pressure, stroke volume, cerebral blood flow velocity and total peripheral resistance (*p* < 0.0054), in the COLD compared to ROOM conditions.

**Conclusions:**

In healthy controls, bolus cold water drinking results in favourable orthostatic cardiovascular responses during HUT/LBNP without significantly altering OT. Using a cold water bolus may result in additional benefits in patients with orthostatic intolerance above those conferred by bolus water at room temperature (by ameliorating orthostatic tachycardia and enhancing vascular resistance responses). Further research in patients with orthostatic intolerance is warranted.

## Introduction

Orthostatic presyncopal symptoms and syncope are common, even in apparently healthy people. Bolus water drinking, at room temperature, has been shown to improve orthostatic tolerance (OT) in healthy participants [1] and in patients with recurrent vasovagal syncope [2], postural tachycardia syndrome (POTS) [3], orthostatic hypotension [4] and spinal cord injury [5]. Drinking water has also been associated with a reduction in vasovagal reactions during whole blood donation [6].

Bolus water drinking improves cerebral blood flow regulation during orthostatic stress accompanied by increased peripheral resistance, with concurrent attenuation of the heart rate (HR) rise and smaller reductions in arterial blood pressure and stroke volume (SV) [1]. These effects are not mediated through correction of dehydration, nor by expansion of plasma volume, but by sympathetic activation. In healthy individuals, water drinking increases muscle sympathetic nerve activity [7] and venous plasma norepinephrine concentrations [8], suggestive of activation of postganglionic adrenergic neurons [9]. The sympathetic modulation of bolus water drinking is thought to be mediated by gastric distension [10] accompanied by activation of osmoreceptors [11] or sodium sensitive afferent nerve fibres [12].

Acute water ingestion is recommended in patients with syncope caused by neurogenic OH in American College of Cardiology/American Heart Association/Heart Rhythm Society (ACC/AHA/HRS) guidelines [13]. Rapid ingestion of ‘cool water’ is an acknowledged treatment strategy in European syncope guidelines for the management of orthostatic intolerance and post-prandial hypotension [14]. However, whether water temperature influences sympathetic responses to an ingested water bolus or affects orthostatic tolerance is less clear. Only one prior study examined this possibility, in four patients with autonomic failure, in whom pressor responses to ingestion of water at 9 °C and 24 °C were similar [8]. The authors of this study did not test responses over a wider temperature range, nor did they examine the impact on OT, or test responses in neurologically intact controls. The splanchnic circulation is a key site for regulation of vascular resistance and capacitance, which are crucial for blood pressure control [11]. It may be that cold-induced vasoconstriction or warm-induced dilation in the gastrointestinal vessels secondary to water ingestion impact both the pressor response and OT.

The primary aim of this study was to assess whether differing water temperatures would alter time to presyncope during incremental orthostatic stress in healthy participants. Our secondary aim was to identify whether differing water temperatures impacted the cardiovascular responses to orthostatic stress. We hypothesised that: (1) the cold water condition would increase OT (time to presyncope) and attenuate the deterioration of cardiovascular and cerebrovascular stability during orthostatic stress in comparison to bolus water drinking at room temperature; (2) the warm water condition would decrease OT and exacerbate the deterioration of cardiovascular and cerebrovascular stability during orthostatic stress in comparison to bolus water drinking at room temperature.

Ethical approval was obtained from the Simon Fraser University Research Ethics Board. All participants provided written informed consent and all experiments were conducted in accordance with the Declaration of Helsinki of 1964 and subsequent amendments.

## Methods

### Participants

Participants were asked to eat only a light breakfast, avoid caffeine, and avoid strenuous exercise for at least 12 h prior to testing. They were also asked to provide a urine sample prior to testing. Urinary sodium concentration was estimated by measuring urinary chloride concentration (Quantab Chloride test strips, 300–6000 mg/L; Hach Canada, London, ON, Canada), with correction for spot sample measurement as detailed by Heeney et al. [15]. Urine specific gravity was measured using Chemstrips 10 test strips (Roche Diagnostics; Laval, QC, Canada). Participants were included if they were aged 19–50 years and able to communicate in English. Participants were excluded if they were pregnant, trying to conceive, or if they had a prior history of cardiovascular or neurological disease.

### Water intervention

**Fig. 1:**
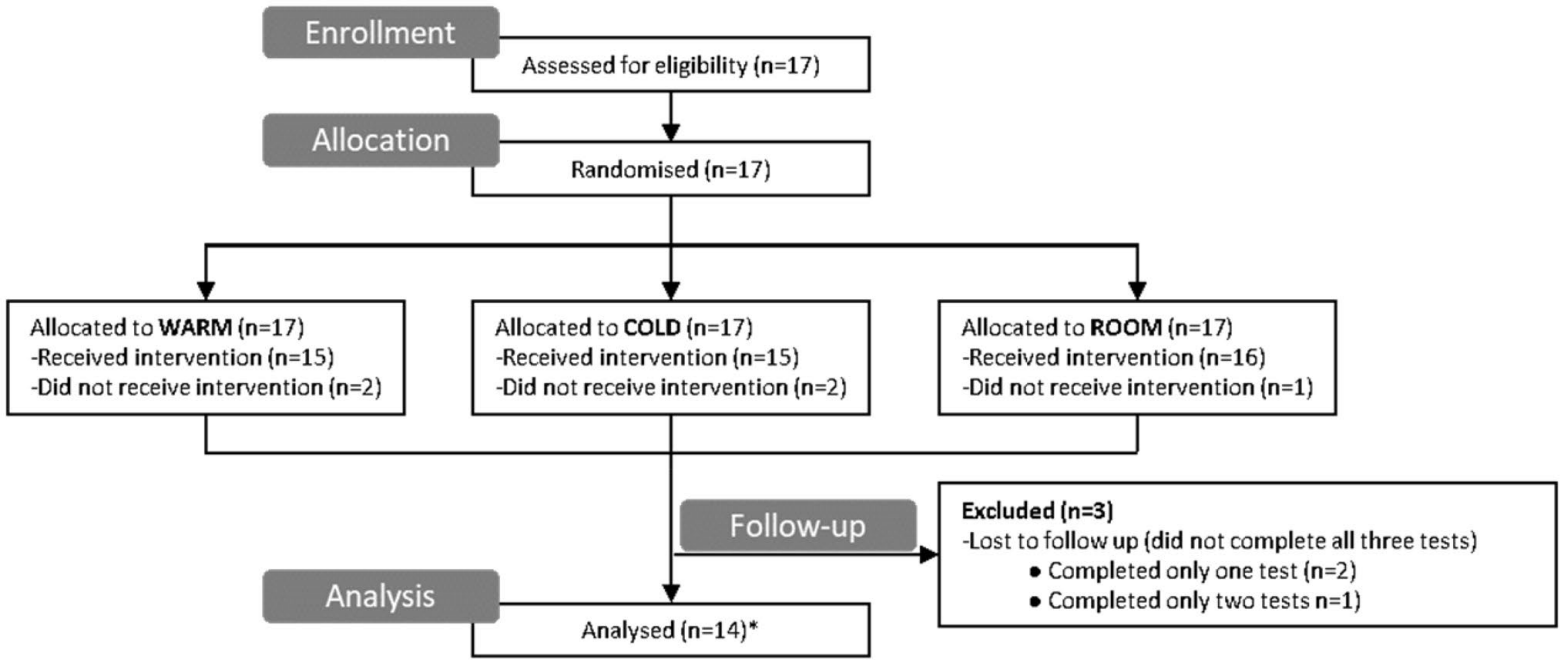
CONSORT flow diagram for enrolment, allocation, follow-up and analysis. Participants were allocated to the three study interventions (ROOM, COLD, WARM; see text for further details) in a randomised controlled crossover design. Data from 14 participants who completed all three components of the study were included in the analysis

The participant recruitment process and randomisation sequence are shown in Fig. 1. Participants attended the cardiovascular physiology laboratory at Simon Fraser University for testing on three separate days. On each test day participants were asked to drink 500 mL of water 15 min prior to the OT test. Participants were randomised (using an online randomisation tool), in single-blind crossover fashion, to either drinking a 500 mL water bolus at room temperature, which was our control condition (20 °C [ROOM]), a 500 mL bolus of ice-cold water (0–3 °C [COLD]) or a 500 mL bolus of warm water (45 °C [WARM]).

### Orthostatic tolerance

OT was measured using head-up tilt (HUT) tests with lower body negative pressure (LBNP). We, and others, have previously shown this technique to be reproducible and reliable, and to have high sensitivity and specificity for differentiating persons with differing OT and for examining the effects of interventions aimed at improving OT [1, 2, 16–23]. Participants rested in the supine position on the tilt table for 15 min to assess baseline cardiovascular parameters, prior to tilting to 15° to facilitate consumption of the water (pre/post-bolus). Participants were asked to consume the entire volume of water (500 mL) within 2 min before being returned to the supine position for a further 15 min of continuous monitoring. The cardiovascular effects of bolus water drinking have been previously shown to occur within 10 min, to peak after 20–40 min, but to dissipate by 90 min [1, 2]. Following this second 15 min in the supine position, HUT was performed at time zero to an angle of 60º for 20 min. After this, while the participant was still tilted, LBNP was applied below the level of the iliac crest at − 20 mmHg for 10 min and then incrementally increased to − 40 mmHg and − 60 mmHg at 10-min intervals. The test was terminated by a blinded investigator at presyncope, defined as a systolic arterial pressure (SAP) < 80 mmHg, or a HR increase > 170 bpm, with symptoms of presyncope (e.g. nausea, light-headedness, tunnel-vision, warmth and perspiration). OT was defined as the time, in minutes, to presyncope.

### Cardiovascular and cerebrovascular monitoring

Non-invasive blood pressure was monitored continuously using the Finometer Pro^™^ (Finometer; Finapres Medical Systems, Amsterdam, The Netherlands). Continuous measures of SAP, diastolic arterial pressure (DAP) and mean arterial pressure (MAP) were obtained. Estimates of SV and cardiac output (CO) were obtained using the Modelflow™ algorithm [24]. HR and rhythm were monitored using a 3-lead electrocardiogram (Finapres ECG Module; Finapres Medical Systems). The total peripheral resistance (TPR) was derived from the MAP divided by CO. Brachial blood flow velocity was measured continuously and non-invasively using an 8-MHz probe (DWL Doppler-Box; Compumedics, Hamburg, Germany) held in place with an adjustable clamp so the angle of insonation remained constant, with the arm supported at heart level. Forearm vascular resistance (FVR) was calculated as the ratio of MAP and brachial blood flow velocity. Middle cerebral artery mean blood flow velocity (CBFV) was measured continuously through the transtemporal window using a 2-MHz ultrasound probe (DWL Doppler-Box; Compumedics), which was fixed in position using a headband to maintain a constant angle of insonation. The depths for the two ultrasound signals were optimised on day 1 and noted, with the same depths used to guide signal acquisition on subsequent tests. Cerebral mean arterial pressure (CMAP) was calculated as: MAP (in mmHg) − (height difference between the transtemporal window and the apex of the heart [cm]/1.36) [25]. Static cerebral autoregulation was determined from the correlation and gradient describing the relationship between CBFV and CMAP during the orthostatic stress test (excluding values at presyncope that would be expected to exceed the lower limit for autoregulation). An increased correlation indicates that CBFV passively follows fluctuations in pressure, suggesting impaired autoregulation. An increased gradient indicates that small changes in pressure elicit large changes in flow, again suggesting impaired autoregulation [1]. End-tidal CO_2_ was recorded continuously using an infra-red analyser (O2Cap; Oxigraf Inc., Mountain View, CA, USA). All cardiovascular recordings were sampled at 1 kHz using an analog-to-digital converter (Powerlab 16/30; AD instruments, Colorado Springs, CO, USA).

### HR and blood pressure variability

Monovariate autoregressive spectral analysis of successive R-R intervals (RRI) were performed during both supine phases (pre/post-bolus drink), as well as during the tilted phase of the test. Data were extracted for analysis over 10 consecutive minutes during steady-state conditions, from 2 to 12 min of each phase. The high-frequency (HF) peak (0.15–0.3 Hz) was identified using computation of the residuals for each spectrum and taken as a marker of cardiac vagal tone (HF RRI). Similar time series were generated for consecutive beat-to-beat SAP, and the low-frequency (LF) peak (0.05–0.15 Hz) was taken as a marker of vascular sympathetic tone (LF SAP) [26, 27]. The gain of the cardiac baroreflex response (cBRS) was obtained through simultaneous spectral analysis of the spontaneous variabilities of RRI and SAP (α index) [28].

### Echocardiography

Transthoracic echocardiography (TTE) was performed by a blinded investigator accredited by the British Society of Echocardiography, using a Philips CX50 ultrasound machine (Phillips, Amsterdam, the Netherlands). A measure of inferior vena cava diameter (IVCd) was performed with the participant supine, prior to water ingestion. The left ventricular outflow tract (LVOT) cross-sectional area (πr^2^; in cm^2^) was determined by a TTE measurement of the LVOT in the para-sternal long-axis view. Left ventricular SV was then calculated from the product of the velocity–time integral (VTI; in cm) of the pulsed-wave Doppler in the LVOT in apical 5-chamber view and the LVOT cross-sectional area. The maximum velocity was also recorded. Repeated measures of SV were obtained at prespecified intervals: following water ingestion; at HUT; 10 min into HUT; and on commencement, and halfway through, each gradation of LBNP. A measure was taken as close as feasibly possible to presyncope. CO was calculated by multiplying the SV (VTI × LVOT cross-sectional area) by the HR.

### Data and statistical analyses

The repeatability of HUT/LBNP is 1.1 ± 0.6 min [21]. We performed an estimate of the required sample size to detect a modest difference in means (effect size *f* = 0.33) using a repeated-measures analysis of variance (ANOVA) with a power of 0.8 and significance level of < 0.05, which returned a recommended sample size of 17 individuals [29].

Supine cardiovascular data are represented as the mean values over the final 2 min of each supine period (pre/post-bolus drink). During HUT and LBNP, cardiovascular data were averaged over the final 30 s of every 2-min interval. LBNP data were presented for the first phase of LBNP only (− 20 mmHg for 10 min) due to the loss of participants to presyncope at higher levels of LBNP. Data are presented as the mean ± standard error of the mean. Measures were assessed for normality using the Shapiro–Wilk test prior to data analysis. Data that were not normally distributed were log-transformed prior to statistical analysis. The IVCd and urinary sodium and urinary specific gravity were compared between the three conditions using a repeated measures one-way ANOVA. Supine cardiovascular data (prior to and post-bolus water) were analysed using a repeated measures two-way ANOVA comparing each condition pre water bolus and post water bolus, with correction for multiple comparisons to give adjusted *p* values (Holm–Šidák). OT, and the correlation coefficient and gradient describing cerebral autoregulation, for the COLD and HOT conditions were compared to those for the ROOM condition using a repeated-measures one-way ANOVA with Dunnett’s multiple comparisons test to give adjusted *p* values. Cardiovascular measures were plotted over time and then compared between HUT/LBNP conditions using a repeated-measures two-way ANOVA (mixed effects model to allow for variable withdrawal), with ROOM used as the control variable and Dunnett’s multiple comparisons test to give adjusted* p* values. The *α* level was set to 0.05. All statistical analyses were performed using GraphPad Prism 8.0 (GraphPad Software, San Diego, CA, USA).

## Results

### Demographics

We recruited 17 participants, of whom 14 completed all three tests of OT. The participants (7 women, 7 men) had a mean age of 25 ± 2 years, mean height of 172 ± 3 cm and mean weight of 69 ± 4 kg. Of the seven women tested, one was using oral contraceptives (with all tests conducted during the active phase), one woman had a hormonal intrauterine device and four were naturally cycling. For the four naturally cycling women, all three tests were conducted during the same menstrual phase (1 in the follicular phase, 3 in the luteal phase). One participant was taking Isotretinoin (brand name: Accutane) and one participant was taking methylphenidate (brand name: Ritalin and Concerta), but did not take them on the 3 testing days.

Comparison of the three conditions showed that there were no significant differences in IVCd (COLD 2.0 ± 0.4 cm; ROOM 2.0 ± 0.1 cm; WARM 2.0 ± 0.2 cm; *p* > 0.9999), urinary specific gravity (COLD; 1.013 ± 0.002, ROOM; 1.017 ± 0.002, WARM; 1.014 ± 0.002; *p* = 0.1568) or urinary sodium (COLD; 119 ± 5 mmol, ROOM; 110 ± 6 mmol WARM; 119 ± 10 mmol; *p* = 0.1585) between conditions.

### Impact of bolus water drinking in the supine position

**Fig. 2:**
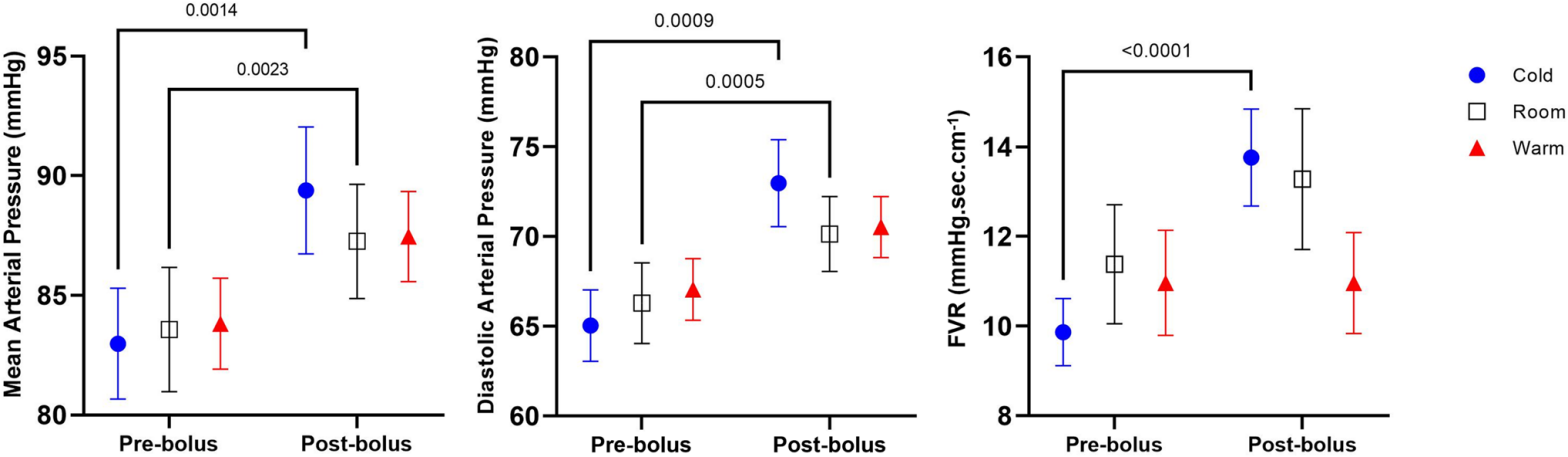
Significant (*p < 0.05) differences in cardiovascular parameters between the ROOM, WARM and COLD conditions in the supine position prior to, and following, ingestion of water bolus, for mean arterial pressure, diastolic arterial pressure and forearm vascular resistance (*FVR*)

Supine post-bolus means were greater for DAP, MAP and FVR in the COLD condition (*p* < 0.0014), and for DAP and MAP in the ROOM condition (*p* < 0.0023), compared to pre-bolus (Table 1; Fig. 2). Supine LF SAP was increased after bolus water drinking compared to pre-bolus in the COLD condition only (*p* = 0.042). Supine HF RRI increased after water drinking only in the COLD condition (adjusted *p* < 0.001) and was significantly larger after drinking in the COLD condition than in both the ROOM (*p* = 0.009) and WARM (*p* < 0.001) conditions. There were no significant differences in SAP, SV, CO, TPR, CBFV, LVOT maximum velocity, cBRS or end tidal CO_2_ between pre and post-bolus water drinking in any condition (*p* > 0.05) (Table 1; Fig. 1).

**Table 1:** Cardiovascular measures for each water temperature condition at pre water bolus and post water bolus, in the supine position prior to head-up tilt

	Water bolus temperature		
	COLD condition	ROOM condition	WARM condition
**Finometer derived values**			
Systolic Blood Pressure (mmHg)			
Pre-bolus	118.9 ± 3.4	118.2 ± 3.4	117.4 ± 2.7
Post-bolus	122.2 ± 4.1	121.5 ± 3.4	121.3 ± 2.8
Diastolic blood pressure (mmHg)			
Pre-bolus	65.0 ± 2.0	66.3 ± 2.3	67.1 ± 1.7
Post-bolus	73.0 ± 2.4*	70.1 ± 2.1*	70.5 ± 1.7
Mean arterial pressure (mmHg)			
Pre-bolus	83.0 ± 2.3	83.6 ± 2.6	83.8 ± 1.9
Post-bolus	89.4 ± 2.7*	87.3 ± 2.4*	87.5 ± 1.9
Heart rate (bpm)			
Pre-bolus	56.8 ± 2.1	59.5 ± 2.2	59.4 ± 2.6
Post-bolus	55.1 ± 1.9	58.7 ± 2.1	59.8 ± 2.5
Stroke volume (mL)			
Pre-bolus	95.9 ± 7.3	91.6 ± 5.6	91.6 ± 5.3
Post-bolus	95.8 ± 8.7	94.7 ± 5.5	92.7 ± 5.5
Cardiac output (L.min^−1^)			
Pre-bolus	5.3 ± 0.3	5.4 ± 0.3	5.4 ± 0.3
Post-bolus	5.2 ± 0.4	5.5 ± 0.3	5.5 ± 0.3
Total peripheral resistance (mmHg.min.L^−1^)			
Pre-bolus	16.3 ± 0.8	16.1 ± 0.8	16.3 ± 0.8
Post-bolus	19.4 ± 2.3	16.6 ± 0.9	16.7 ± 0.8
LF SAP (mmHg^2^)			
Pre-bolus	2.8 ± 0.6	3.5 ± 0.6	3.1 ± 0.4
Post-bolus	4.5 ± 1.1*	3.6 ± 0.6	3.8 ± 1.2
HF RRI (ms^2^)			
Pre-bolus	920 ± 457	476 ± 128	626 ± 330
Post-bolus	1298 ± 459*	766 ± 301^†^	653 ± 302^†^
BRS α-index (ms.mmHg^−1^)			
Pre-bolus	25.4 ± 6.0	22.2 ± 3.9	22.1 ± 4.5
Post-bolus	25.6 ± 4.1	17.4 ± 2.6	17.6 ± 3.7
**Echocardiography derived values**			
Stroke volume (ml)			
Pre-bolus	83.2 ± 5.9	78.9 ± 4.8	76.7 ± 4.1
Post-bolus	84.8 ± 5.9	81.3 ± 4.5	79.9 ± 4.7
LVOT max velocity (m.s^−1^)			
Pre-bolus	90.3 ± 3.9	89.0 ± 3.2	88.9 ± 3.7
Post-bolus	88.7 ± 4.9	88.7 ± 4.9	89.5 ± 3.1
Cardiac output (L.min^−1^)			
Pre-bolus	4.6 ± 0.3	4.6 ± 0.2	4.6 ± 0.3
Post-bolus	4.8 ± 0.4	4.7 ± 0.3	4.6 ± 0.3
**Cerebral and forearm Doppler derived values**			
Forearm vascular resistance (mmHg.sec.cm^−1^)			
Pre-bolus	9.9 ± 0.8	11.4 ± 1.3	11.0 ± 1.2
Post-bolus	13.8 ± 1.1*	13.3 ± 1.6	11.0 ± 1.1
Cerebral blood flow velocity (cm.s^−1^)			
Pre-bolus	69.0 ± 4.1	61.6 ± 5.1	61.6 ± 5.6
Post-bolus	67.6 ± 4.7	62.4 ± 5.1	60.9 ± 5.9

Values in table are the mean ± standard error of mean (SEM)

*BRS* Baroreceptor,* HF RRI* high-frequency R-R interval, *LF SAP* low-frequency systolic arterial pressure, *LVOT* left ventricular outflow tract

*Significant difference from pre-bolus in the same condition; ^**†**^significant difference from COLD condition in the same test phase

### Impact of bolus water drinking on responses to orthostatic stress

Our analysis did not find any significant difference in OT in the COLD (33 ± 3 min; *p* = 0.3321) and WARM (32 ± 3 min; *p* = 0.6764) conditions in comparison to the ROOM condition (31 ± 3 min).

In the COLD condition, SAP, SV, CBFV and TPR were significantly greater during orthostatic stress (Table 2; Fig. 2), with significant decreases in HR and CO, compared to the ROOM condition (*p* < 0.05). The increase in FVR in the COLD condition compared to the ROOM condition did not quite reach statistical significance (*p* = 0.059). The maximum HR during the orthostatic stress tended to be lower in the COLD condition (119 ± 2 bpm; *p* = 0.05) compared to the ROOM condition (128 ± 4 bpm), although this did not quite reach statistical significance. SV inferred from echocardiography measures was increased in the COLD condition compared to the ROOM condition (*p* = 0.0004); however, echocardiographic measures of CO were not significantly different between the COLD and ROOM conditions. During tilt, HF RRI was significantly increased in the COLD compared to the ROOM condition (*p* = 0.003).

**Table 2:** Physiological measures for each water temperature condition during head-up tilt/lower body negative pressure

Physiological measures^a^	Water bolus temperature			*p* value (adjusted)^b^	
	COLD condition	ROOM condition	WARM condition	COLD vs. ROOM	WARM vs. ROOM
**Finometer-derived values**					
Systolic blood pressure (mmHg)	122.1 ± 3.0	118.8 ± 2.9	118.8 ± 2.6	0.0124*	0.8101
Diastolic blood pressure (mmHg)	80.4 ± 0.3	79.4 ± 0.4	80.1 ± 0.4	0.7248	0.8453
Mean arterial pressure (mmHg)	95.4 ± 0.5	93.7 ± 0.7	94.0 ± 0.3	0.2187	0.9973
Heart rate (bpm)	73.2 ± 2.5	83.1 ± 3.0	80.8 ± 2.4	< 0.0001*	0.0280*
Stroke volume (mL)	69.5 ± 2.3	63.2 ± 8.6	64.5 ± 2.3	< 0.0001*	0.3360
Cardiac output (L.min^−1^)	4.9 ± 0.03	5.1 ± 0.04	5.1 ± 0.05	0.0073*	0.9855
Total peripheral resistance (mmHg. min.L^−1^)	20.9 ± 0.5	19.3 ± 0.1	19.3 ± 0.2	0.0054*	0.9858
LF SAP (mmHg^2^)	14.9 ± 3.4	16.0 + 3.1	16.5 ± 2.6	0.892	0.756
HF RRI (ms^2^)	360 ± 126	156 ± 39	228 ± 70	0.003*	0.367
BRS α-index (ms. mmHg^−1^)	10.4 ± 1.6	8.3 ± 0.8	9.1 ± 0.9	0.964	0.870
**Echocardiography-derived values**					
Stroke volume (mL)	59.5 ± 3.1	53.2 ± 3.3	56.4 ± 2.8	0.0004*	0.1485
LVOT max velocity (m.s^−1^)	84.6 ± 2.1	83.7 ± 1.3	89.2 ± 1.9	0.8423	0.0583
Cardiac output (L.min^−1^)	4.6 ± 0.2	4.7 ± 0.1	4.9 ± 0.2	0.2263	0.3883
**Cerebral and forearm Doppler-derived value**s					
Forearm vascular resistance (mmHg. s .cm^−1^)	21.1 ± 0.7	19.4 ± 0.4	17.6 ± 1.0	0.0588	0.0747
Cerebral blood flow velocity (cm.s^−1^)	43.2 ± 1.8	36.3 ± 1.8	41.3 ± 1.4	< 0.0001*	< 0.0001*

Values in table are the mean ± SEM

*Statistically significant difference compared to ROOM condition at *p* < 0.05

^a^LF SAP, HF RRI and BRS α-index were determined during head-up tilt alone, to ensure analyses were conducted during steady-state conditions

^b^Statistical comparisons were made with ROOM condition as the baseline condition

**Fig. 3:**
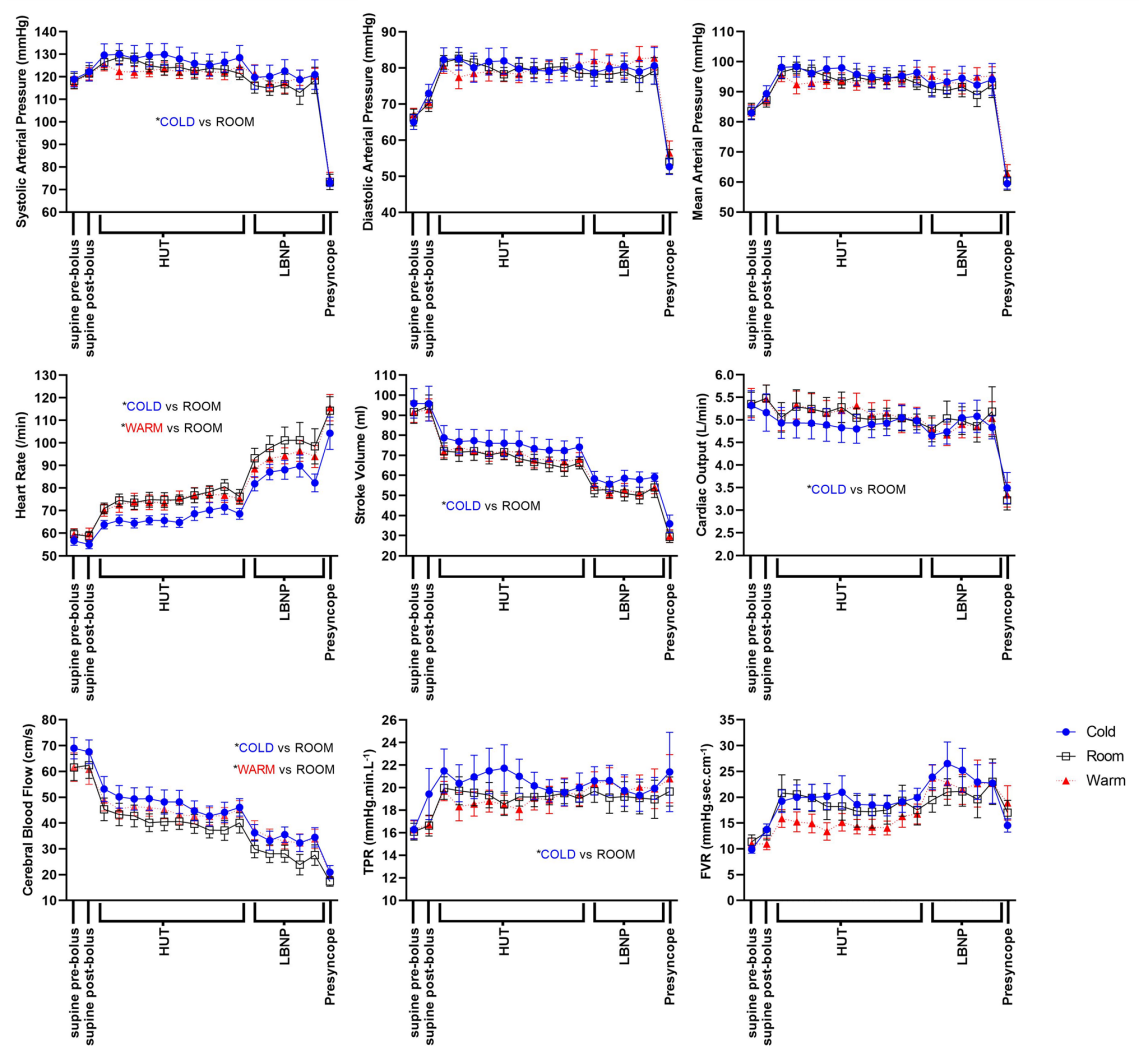
Mean cardiovascular and cerebrovascular parameters plotted over time for the COLD, ROOM and WARM conditions. Heart rate, stroke volume, systolic blood pressure, total peripheral resistance and cerebral blood flow were all significantly different in the COLD condition, for HUT and LBNP, in comparison to the ROOM condition. Asterisk (*) denotes significant difference after adjustment for repeated measures

In the WARM condition there were significant increases in CBFV and decreases in HR compared to the ROOM condition (both *p* < 0.05) (Table 2; Fig. 3). The trend for an increase in LVOT velocity and decrease in FVR in the WARM condition compared to the ROOM condition did not reach statistical significance.

There were no significant differences in end tidal CO_2_ between the ROOM and WARM conditions, and between the ROOM and COLD conditions (COLD: 32.3 ± 0.50 mmHg; ROOM: 32.3 ± 1.9 mmHg; WARM: 32.3 ± 1.2 mmHg; *p* > 0.7949). There were also no significant effects of water temperature on either the correlation coefficient (*p* = 0.174) or the gradient (*p* = 0.082) describing the relationship between CMAP and CBFV between these conditions. Post-hoc comparisons showed that the gradient tended towards being less steep, indicating better autoregulation, in the COLD versus ROOM comparison, but this did not reach criteria for statistical significance (*p* = 0.071). cBRS was not different between the COLD and ROOM conditions or between the WARM and ROOM conditions.

## Discussion

To our knowledge this is the first study to assess the effect of the temperature of an ingested water bolus on OT. Our results show that there are significantly different cardiovascular and cerebrovascular responses to orthostatic stress following the consumption of a 500 mL water bolus at different temperatures. Although OT was not increased in the COLD condition, we found that orthostatic cardiovascular control was significantly improved compared to the ROOM condition. Increased SV, SAP and CBFV were achieved through enhanced vascular resistance responses (TPR) and increases in vascular sympathetic tone (LF SAP), with attenuated HR responses accompanied by increases in cardiac vagal tone (HF RRI), reflecting an attenuation in HR rise and improvement in cardiac reserve. A comparison of the WARM and ROOM conditions revealed that in the WARM condition there was a significantly decreased orthostatic HR and a significantly increased CBFV. OT did not differ in participants between the WARM and ROOM conditions.

Bolus water drinking, typically 500 mL, has been shown, in several studies, to improve OT both in healthy younger participants as well as in patients with syncope and those with autonomic dysfunction or autonomic failure [1–5, 8, 30, 31]. These studies have consistently shown an attenuation of the HR rise and SV reduction during HUT [1] and, for the most part, have shown a significantly increased blood pressure, TPR and CBFV in comparisons of a 50 and 500 mL bolus [1, 2]. The mechanism underlying how bolus water drinking improves OT is less clear, although the improvement is likely to be sympathetically mediated [1, 7–9, 32], which is compatible with our findings showing increases in markers of vascular sympathetic tone and peripheral vasoconstriction. However, in participants with sympathetic denervation due to autonomic failure, the pressor response still occurs, potentially suggesting an alternate or dual mechanism [33]. Gastric distention due to the increased volume is thought to be initially responsible for the improvement in cardiovascular stability during HUT [10]; however, these effects are likely to diminish over time as the water transits into the small and large bowel. More enduring effects may be related to the hypotonicity of the water, as similar improvements in cardiovascular stability are not seen with an ingested saline solution [11, 34]. A murine study in mice with baroreflex failure (baroreflex deafferentation) indicated a role of the osmosensitive transient receptor potential vanilloid 4 channel in mediating sympathetic activation [11].

Nonetheless, the accepted paradigm has been that the temperature of the consumed water makes no difference to cardiovascular parameters [8], which is contrary to our findings. One previous study [32] comparing the consumption of cold water (3 °C) and water at room temperature, at rest and without orthostatic stress, found that there was a significantly decreased HR with cold water drinking. The mechanism described was an increase in vagal tone, perhaps through activation of thermosensitive afferent vagal nerve fibres in the oesophagus, stomach and duodenum [32, 35]. Our data also support cardiac vagal activation with cold water drinking, and in support of previous observations, this could not be explained by alterations in cBRS. The HR increases during the initial phases of tilting are thought to be primarily due to a baroreflex-mediated withdrawal of parasympathetic tone [36, 37]; consequently, a vagally-mediated maintenance of parasympathetic tone in the COLD condition could explain the comparatively lower HR during orthostatic stress. With a lower HR, diastole is longer resulting in a longer filling time for the left ventricle and subsequent increased SV, particularly during HUT/LBNP. This is likely to be coupled with decreased venous capacitance. Given the significant mean reduction in HR observed in the COLD condition in this study (10 bpm) compared to the ROOM condition, there is a potential role for cold water drinking to ameliorate the orthostatic tachycardia and low stroke volumes that are the hallmark features of orthostatic intolerance in patients with POTS [38].

While the augmented vagal tone might explain the cardiac responses observed in the COLD condition, it would not explain the enhanced vascular responses observed. We showed that measures of supine vascular resistance and sympathetic vascular tone increased following ingestion of a cold water bolus, and that resistance responses remained increased during HUT, with an associated increase in SAP throughout the test, compared to the ROOM condition. These findings suggest that cold water bolus drinking increases sympathetic responses compared to drinking a water bolus at room temperature or at warm temperature. Cold water may induce sympathetically-mediated vasoconstriction of abdominal viscera, resulting in a greater venous pressure in the splanchnic system, and reduced venous capacitance, ultimately resulting in an increased SV, which would be expected to reflexively increase vagal tone and further contribute to the lower HR observed. We could not evaluate whether there was an increase in IVCd after cold water drinking, which might support this hypothesis, because we only estimated this parameter prior to the ingestion of water. In retrospect, a measure following the ingestion of the water bolus would have been useful. Regardless of the mechanisms, the enhancement of vascular resistance responses in the COLD condition might provide additional benefit to patients with disorders of OT in whom vascular resistance responses are impaired [18]. Of note, while studies have demonstrated a limited relationship between the proxy for sympathetic vascular tone we used, LF SAP, and muscle sympathetic nerve activity at low levels of sympathetic stimulation [39], the relationship does become more robust with sympathetic activation. In addition, LF SAP is well correlated with plasma noradrenaline, suggesting that it is a reasonable proxy for end organ sympathetic responses during the strong sympathetic activation of orthostatic stress [26].

When comparing the COLD condition to the ROOM condition, the orthostatic CBFV was significantly increased. This has important implications because cerebral hypoperfusion is the final common pathway associated with syncope and the development of presyncopal symptoms, and cognitive impairment and brain fog are frequently associated with the cerebral hypoperfusion that accompanies orthostatic intolerance [40, 41]. Improvements in CBFV, therefore, have the potential to significantly improve quality of life for those with orthostatic syncope and presyncope. One prior study on the impact of cold water ingestion on cerebral blood flow showed a significant increase in comparison to ingestion of water at room temperature (22 °C), which was thought to be due to parasympathetic activation of the trigeminal, vagal and glossopharyngeal nerves, as well as the effect of sympathetic activation on cardiovascular physiology [42]. We observed no additional significant effect on autoregulation based on the water temperature, implying that the enhancement in CBFV reflects a higher perfusion pressure rather than a shift in autoregulation.

Based on these favourable cardiovascular responses in the context of orthostatic control, we would have expected OT to be improved in the COLD condition as compared to the ROOM condition. This study was potentially under-powered to detect a change in OT in healthy participants with normal orthostatic cardiovascular reflex responses; however, our retrospective power was quite strong (0.7), even after accounting for the reduction in our sample size due to some participants not completing all three tests. The cardiovascular responses of decreased CO (decreased HR but increased SV), with increased TPR, may have attenuated the blood pressure rise in the COLD condition, and this has been described previously in healthy participants [11]. It is also possible that there was a ceiling effect imposed from testing healthy controls with robust orthostatic responses. A repeat study performed in individuals with orthostatic syncope or those with autonomic dysfunction may well yield a demonstrable change in OT or improved symptomatology with bolus cold water ingestion given the beneficial cardiovascular and cerebrovascular responses observed in these healthy controls.

We hypothesised that cardiovascular responses and OT would be impaired in the WARM condition. However, this was not the case and, in fact, there was also a reduced HR when comparing the WARM condition to the ROOM condition. In the absence of tilt these changes have not been previously demonstrated [8]. We also saw a significant increase in CBFV in the WARM condition in comparison to the ROOM condition, which mirrors the changes seen in the COLD condition. The similar responses with the disparate COLD and WARM water stimuli are a challenge to reconcile without further detailed investigations. However, the findings of a previous study may provide some indication: participants given 350 mL of coffee or water, both at 40 °C, showed a small increase in CBFV in the water arm only (53.4 ± 10.1 to 55.4 ± 10.0 cm/s) although the difference was not significant [43].

As the effect of bolus water drinking has been studied several times [1, 2], we opted not to perform a baseline HUT without water bolus. This served to reduce the participant burden for this study, which still involved multiple tests of OT, and to reduce overall redundancy of the experimental design. However, it did mean that we were unable to determine whether there were differences in the responses to water temperature relative to the baseline condition, or whether there would have been larger improvements in OT in the COLD condition in those with a lower baseline OT. To put this into context, it has already been shown, with the same protocol as used in the present study, that drinking 500 mL of water at room temperature increases OT in healthy controls by 5 ± 1 min compared to placebo (*p* < 0.001) [1].

We opted to wait 15 min following bolus water ingestion before the HUT test, as in previous studies [1, 2], because the peak blood pressure response to water ingestion is reported to occur after 20–30 min, and to last for 60 min; consequently, the effects of water ingestion would be maximal during the orthostatic portion of the test [30, 44]. Of note, the vagally-mediated reductions in HR and subsequent increase in SV occur early following cold water ingestion and persist for approximately 45 min [30]. As the present study evaluated healthy controls, and all participants in all three conditions had an intervention which is known to lengthen OT, it is possible that the relatively long test duration attenuated the cardiovascular responses towards the end of the test in the COLD condition, resulting in no significant difference in OT. It is possible that more robust responses may be observed with a cold water bolus in patients with poor OT.

We utilised multiple methods to ascertain any differences in cardiovascular parameters during orthostatic stress continued until presyncope. We confirmed the relative increase in SV in the COLD condition by both the Modelflow™ algorithm and echocardiography, although no significant increase in CO was seen with echocardiography. The robust increases in SV with cold water ingestion using both methodological approaches confirm that the cardiovascular changes described are not due to errors in measurement; however, echocardiography is known to underestimate SV [45], and this might explain the lower values based on echocardiography compared to Modelflow™.

This study was registered in order to comply with the International Committee of Medical Journal Editors (ICMJE) guidelines. One of its limitations is that the trial registration was performed in retrospect because trial registration was not required for our institutional or ethical guidelines (since the testing of a water-based intervention is not considered a drug, therapeutic or biological device) but rather to meet publication criteria.

Even though our results showed a limited impact of water temperature on the effect of bolus water drinking on OT in these healthy controls, the enhancements in cardiovascular responses to orthostatic stress with cold water drinking would likely provide clinical benefit in certain situations. For example, we anticipate that during single application, in large populations, such as prior to blood donation [46] or military parades [47], the use of cold water may be of additional benefit above that seen by water drinking alone. Patients with POTS, who have excessive HR responses to standing, may benefit from bolus cold water drinking due to the demonstrable effect of cold water drinking in reducing the HR rise during orthostatic stress. In addition, patients with orthostatic intolerance secondary to impaired vascular resistance responses may benefit from the larger vascular resistance responses to orthostatic stress noted with cold water drinking.

## Conclusion

In this study, bolus cold water drinking resulted in favourable cardiovascular responses during HUT/LBNP, without significantly altering OT. It is possible that bolus cold water drinking would result in additional benefits in patients with orthostatic intolerance above that conferred by bolus water drinking at room temperature. Cold water bolus drinking may be of particular use in patients with POTS, due to the significant reduction in HR. Further research in patients with orthostatic intolerance secondary to impaired vascular resistance responses is also warranted given the enhanced vascular resistance responses seen with cold water ingestion.

## Data Availability

Data available on request.

## Declarations

### Registration

ISRCTN14714572.
